# Slow, stochastic transgene repression with properties of a timer

**DOI:** 10.1186/gb-2006-7-6-r47

**Published:** 2006-06-09

**Authors:** Clifford L Wang, Desirée C Yang, Matthias Wabl

**Affiliations:** 1 Department of Microbiology and Immunology, University of California, San Francisco, CA 94143-0414, USA

## Abstract

**Background:**

When gene expression varies unpredictably between genetically identical organisms, this is sometimes ascribed as stochastic. With the prevalence of retroviral vectors, stochastic repression is often observed and can complicate the interpretation of outcomes. But it may also faithfully reflect characteristics of sites in the genome.

**Results:**

We created and identified several cell clones in which, within a given cell, retroviral transcription of a transgene was repressed heritably and essentially irreversibly. This repression was relatively slow; total repression in all cells took months. We observed the dynamics of repression and found that they were ergodic, that is, tending with a probability to a final state independent of previous conditions. Different positions of the transgene in the genome demonstrated different dynamics. At a position on mouse chromosome 9, repression abided by near perfect first-order kinetics and was highly reproducible, even under conditions where the number of cell generations per day varied.

**Conclusion:**

We propose that such a cell division independent 'off' mechanism could play a role in endogenous gene expression, potentially providing an epigenetically based timer for extended periods.

## Abstract 2

The dynamics of retroviral transgene repression were analyzed in several clones; repression was found to be slow and different genomic positions showed different dynamics.

## Background

When a single outcome is not a certainty, but instead chosen seemingly randomly from two or more possible states, that process is often termed 'stochastic'. Stochasticity is also used to explain phenotypic differences between cells of a genetically identical population. Examples include how individual cells of *Escerichia coli *[1] or yeast [2] in the same culture produce differing amounts of a protein; yeast express either the **a **or α mating type locus [3]; olfactory neurons each express a different odorant receptor [4]; mature T cells choose to express either CD4 or CD8, but not both [5]; B cells express one functional immunoglobulin allele while excluding the other [6,7]. Ronai *et al*. have demonstrated that within a clonal population of cells, epigenetic differences at the immunoglobulin locus can lead to distinct expression states that can be inherited from generation to generation [8-10]. Also, using transgenic reporter constructs, Walters *et al*. studied the effect of enhancers on genetic variegation that results from slow gene repression [11,12]. Weinberger *et al*. [11] have shown that the fluctuations in amounts of the viral protein Tat can lead to different expression states of green fluorescent protein (GFP) expressed from an HIV-based vector [13]. They demonstrated that Tat is a decisive component in a positive feedback loop, and that stochastic and variable expression of Tat affects whether GFP is expressed at a high or low state. Such phenotypic bifurcation may also explain proviral latency [13].

The assumption that a mechanism is stochastic can be reasonable, and in biology many stochastic models abound [2,14-18]. But in biology, final outcomes are also often instructed and so the issue of stochasticity is not always clear. While phenotypic outcomes might appear random, if one tallies enough events, the ensemble of events should reveal that outcomes are probabilistic. We describe a system to characterize the repression of a transgene in a mammalian cell line. Using this system, we demonstrate that slow repression can abide by first-order decay kinetics over long time periods. Here, we focus not on the fluctuation of expression due to stochasticity, but describe how predictable dynamics of repression can be determined by a stochastic decision.

## Results

**Figure 1:**
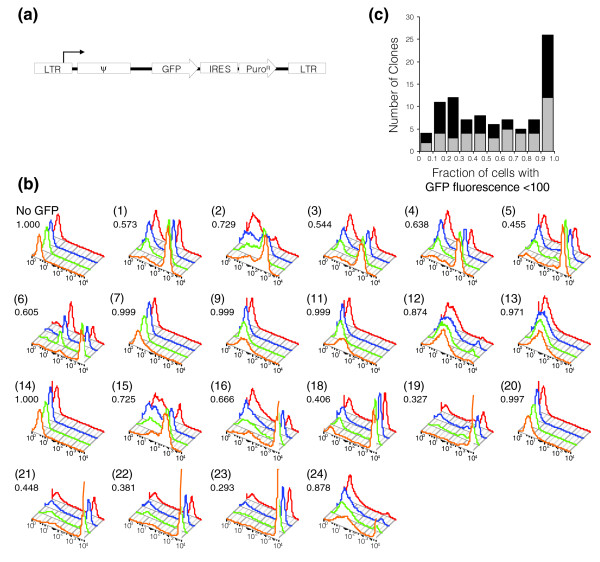
Expression of GFP fluorescence in clones. **(a) **Clones were transduced with a retroviral vector. LTR, long terminal repeat; ψ, retroviral packaging signal; GFP, green fluorescent protein; IRES, internal ribosome entry site; Puro^R^, puromycin resistance gene. **(b) **Flow cytometry profiling GFP fluorescence of untransduced cells (No GFP) and clones 1 to 24, which were transduced with the GFP vector (other clones in Additional data file 4). X-axis, relative fluorescence, where that of untransduced cells was set to 3.0; Y-axis (from front to back), 13, 22, 32, and 42 days in culture; Z-axis, normalized cell number; upper left corners show the clone identification number followed by the fraction of the population with fluorescence less than 100 after 32 days. **(c) **Histogram summary of GFP repression in clones after 32 days. Bars represent number of clones transduced with GFP (gray) and clones transduced with GFP plus an adjacent Ig enhancer (black).

First, using the pre-B cell line 18-81, we created transgenic cell lines that expressed GFP. Cells were infected with a retroviral vector containing GFP (Figure 1a) or GFP followed by an enhancer from the immunoglobulin (Ig) heavy chain. Two days later, we isolated single, infected cells exhibiting fluorescence greater than 100 relative units. Clones were then expanded from these single cells. The infection was at multiplicity of less than 5%. Thus, if one assumes a Poisson distribution of infection, greater than 95% of all the clones are likely to contain only one vector copy. For 10 clones we determined the sites of vector integration (Table 1). None of the 10 contained more than one copy of the vector.

### Gene repression as a state function

Since our aim was to study gene inactivation, cultures were not initially selected with antibiotics. Also, because we waited two days before isolating fluorescent clones, we avoided those clones where GFP inactivation was rapid (that is, occurring in less than two days). This method facilitated the study of gene repression that is observable over longer periods of time. By using flow cytometry to measure GFP fluorescence, we were able to track gene repression of a population on a cell-by-cell basis. In total, we created and tracked 93 clones (Figure 1b; Additional data file 4) that differed in GFP expression. Differences in integration sites likely account for these differences. Of the 93 clones, 45 clones were transduced with GFP and 48 clones were transduced with GFP followed by the Ig enhancer. Between those with and without the additional enhancer, we observed little discernable difference in gene inactivation. Nearly all of the GFP expression profiles had multiple peaks (for example, bi- or tri- modal) and this meant that fluorescence behaved as a state property, with decreases in fluorescence corresponding to transition from a high to low expression state (Figure 1b; Additional data file 4). Out of 93 clones, there were only three (clones 9, 11, and 13) in which the decrease in fluorescence appeared continuous, and all were decreases from an initially low level.

After 32 days in culture, 26 of 93 clones had repressed GFP expression to the point that more than 90% of the cell population produced a relative fluorescence less than 100 (Figure 1c). In contrast, 4 of 93 clones had populations where less than 10% of the cells had fluorescence less than 100. In 38 of the clones, 10% to 50% of the cells in the culture produced fluorescence less than 100.

**Figure 2:**
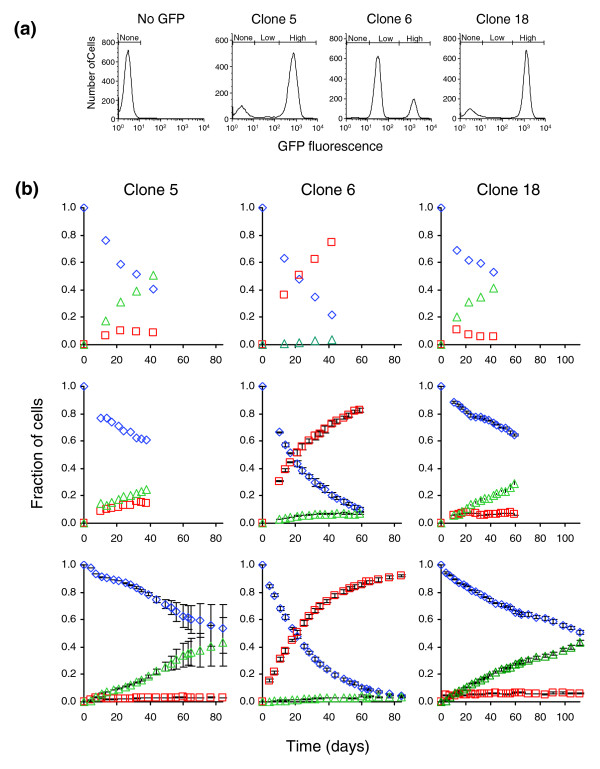
GFP fluorescence of clones 5, 6, and 18. **(a) **Levels of GFP fluorescence, high, low, and none, were defined by flow cytometry profiles of untransduced cells (No GFP) and clones 5, 6, and 18. X-axis, relative fluorescence; Y-axis, number of cells. **(b) **GFP fluorescence: top, during initial expansion from a single cell and subsequent passaging (no error bars because there was only one replicate, that is, *n *= 1); middle, fluorescence in cells isolated from the first experiment (top) (clone 5 without error bars because *n *= 1); bottom, fluorescence in cells isolated from the second experiment (middle). Fraction of cells are shown with high (diamonds), low (squares), or no (triangles) fluorescence.

We selected clones 5, 6 and 18 for further study. All had been transduced with the vector encoding GFP (with no additional Ig enhancer). GFP expression in clones 5 and 18 tended to be an all-or-none phenomenon, with fluorescence being produced either highly or not at all (Figure 2a). In clone 6, most cells expressed GFP at either a high or a low, yet detectable, state, with a small percentage of cells expressing no detectable GFP fluorescence. Since expression of GFP behaved as a state function, we could tally outcomes as being high, low, or no fluorescence (as gated on Figure 2a). When the absolute data were recast in terms of states (Figure 2b; Additional data files 1, 2, 3), one could clearly see how the decrease in cells expressing high GFP coincided with the increase in cells with repressed GFP, that is, those having either low or no fluorescence.

### Stochastic repression of gene transcription

After the first experiment (Figures 1b, and Figure 2b, top), cells with high fluorescence were isolated by fluorescence-activated cell sorting (FACS) and reintroduced to culture. Again, GFP was similarly repressed (Figure 2b, middle). From this second experiment, cells with high fluorescence again were re-isolated and monitored (Figure 2b, bottom), this time for a longer period. Again, the dynamics of repression were similar to the previous experiments. It appeared that the re-isolated populations were not affected by time spent in culture and had no memory of previous experiments. This property was convenient from a practical standpoint - for subsequent experiments, we could reset cultures to a common starting point where all cells expressed high, unrepressed GFP.

**Figure 3:**
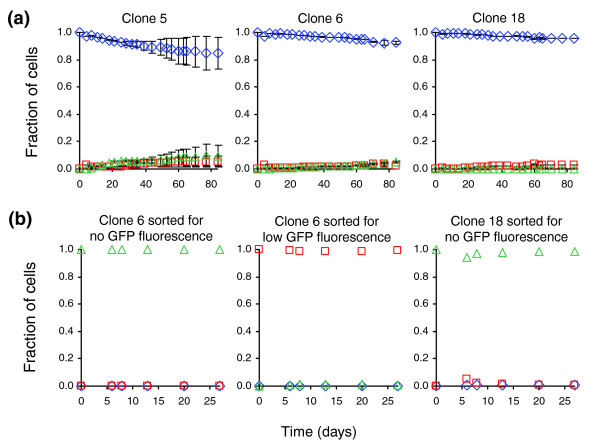
GFP fluorescence of clones 5, 6, and 18 with antibiotic selection or pre-sorted cells with repressed levels of GFP. Levels of GFP fluorescence by **(a) **cultures grown with puromycin and **(b) **cultures started with cells producing low or no fluorescence (no error bars because *n *= 1). Fraction of cells are shown with high (diamonds), low (squares), or no (triangles) fluorescence.

When the cells were re-sorted for high GFP expression and cultivated in medium containing puromycin, less repression occurred (Figure 3a). Selection with puromycin enriched for cells expressing puromycin resistance. In our construct, GFP and puromycin resistance are encoded on the same transcript. Thus, the puromycin-dependent enrichment for cells with high fluorescence indicated that the repression observed without puromycin was caused by a decrease in transcription. Quantitative real time (RT)-PCR, using beta-actin as an internal standard, corroborated this finding. From cultures grown without puromycin, we isolated cells that had or had not repressed GFP expression. Cells from clone 6 producing low GFP fluorescence and those from clone 18 producing undetectable GFP fluorescence contained, respectively, 55 and 21 times less GFP mRNA than their high GFP counterparts (not shown). Since the cell line used in this study undergoes hypermutation [19], we considered the possibility that the changes in transcription resulted from mutations in DNA. We separated clone 6 cells producing high fluorescence from those producing low fluorescence and sequenced the GFP gene. Because the majority of GFP sequences (17 of 22) isolated from cells producing low fluorescence contained no mutations at any base within the coding region, we conclude that mutations in the coding region could not account for all cases of gene repression. The mutation frequency in cells producing high fluorescence was comparable; 15 of 17 sequences contained zero mutations. Though we cannot rule out mutation occurring in the promoter, we think this is unlikely, since such mutation in B cells (caused by endogenous activation-induced cytidine deaminase) is only known to occur in transcribed regions of DNA, at some distance from the promoter [20]. Documented mutation rates [19,21] for this cell line are three or more orders of magnitude lower than the rate of repression observed.

### Heritability and irreversibility of gene repression

For clones 6 and 18, we asked whether the repressed transcription states were stably inherited. When we isolated cells with repressed GFP levels (low or no observable fluorescence) and put these cells back into culture, we saw no high GFP-expressing cells reemerge (Figure 3b). Here we had passaged the cells by splitting the cultures by a third daily (that is, for a 3 ml culture, 2 ml of fresh medium was combined with 1 ml of the old culture) for 27 days. Because cultures reached a steady-state number of cells (stationary phase) each day, the amount that the culture was split corresponded to the number of cell generations per day. By approximating the average number of generations per day to be equal to -ln(split fraction)/ln(2), we determined that repressed expression was inherited through at least 43 generations. This meant that the observed GFP dynamics were due to repression alone and not a combination of repression and de-repression.

### Kinetics of gene repression

We queried whether different proliferation conditions could affect gene repression. Since we needed to maintain healthy viable cells over months, inhibitors of cell cycle to control growth rate were not an experimental option for us. Instead, cultures were 'split' (that is, diluted) daily with different fractions of new medium, ranging from a half to a sixth. For cultures that reached steady-state cell concentrations each day following medium replenishment, we calculated that splitting by a half, a third, a quarter, a fifth, and a sixth corresponded to an average of 1.0, 1.6, 2.0, 2.3, and 2.6 generations, respectively, per day. In a previous study, by staining with carboxyfluorescein diacetate succinimidyl ester (CFSE) we have established that these different culture conditions do vary the number of cell generations per day [22]. In addition, the 18-81 cell line was well suited for these experiments because they are highly non-adherent and divide rapidly. In all conditions we studied here, the cell medium never reached a pH under 7 (as observed by phenol red indicator), exhibited no visible signs of cell stress - lack of vacuoles, consistent cell shape and membrane integrity, and so on - and viable cell staining determined the presence of less than 2% dead cells. We also noted that clone 5 cells could not divide fast enough to keep pace with a culture dilution rate of one-fifth and clone 6 could not do so when diluted by a sixth. Thus, here we have analyzed only cultures where cell numbers were steadily maintained. Although our method to vary the number of cell generations worked well, it is indirect, and our results should be interpreted with this in mind.

**Figure 4:**
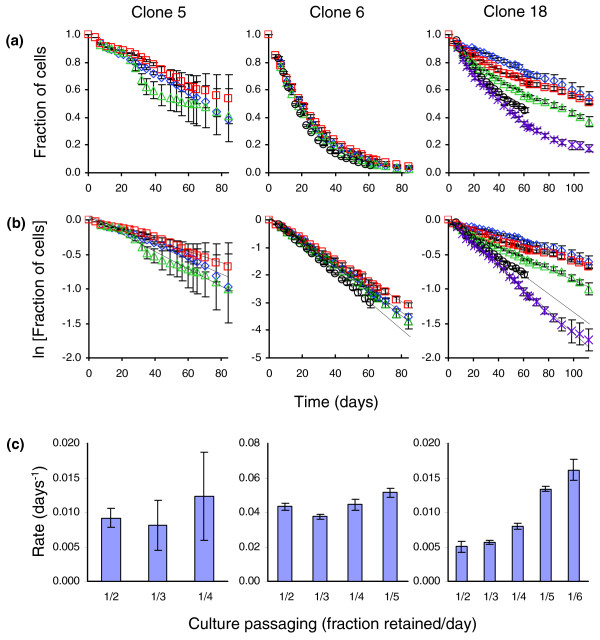
Repression of GFP by clones 5, 6, and 18 with varied culture passaging conditions. **(a) **Fraction of cells with high GFP fluorescence. **(b) **Natural log of the fraction, where the dotted line is fit by linear regression. When passaging cultures daily, different fractions of culture were retained: one-half (diamonds), one-third (squares), one-quarter (triangles), one-fifth (circles), or one-sixth (crosses). **(c) **Repression rates (slope of ln [cell fraction]).

Although we grew all the clones at the same time and tried to maintain identical experimental conditions, with clone 5 the data varied substantially between experiments performed at different times and between replicates performed simultaneously (Figures 2 and 3). This variability in the data precludes statements about kinetics of repression by clone 5. For clones 6 and 18, repression dynamics were more reproducible. The average standard deviations (error bars in Figure 4) between replicate cultures (*n *= 4) for all conditions were 4% and 2% for clones 6 and 18, respectively.

We evaluated the fit of the GFP repression dynamics (Figure 4a) to zero, first, and second-order decay kinetics (Table 2). Here the fraction of cells (c) expressing an unrepressed level of GFP was represented as a function of time (t) and a characteristic rate (k), such that C = 1 - kt (zero order), C = e^-*kt *^(first order), or C = (1 + kt)^-1 ^(second order).

Dynamics of zero order would suggest that gene repression was instructed by some factor unrelated to the cells in the culture, or that there existed some intricate quorum-sensing among cells, so as to maintain a constant repression rate despite the fact that the pool of 'available' cells (that is, with unrepressed GFP) was decreasing with time. As there was no clear case where the gene repression was closer to zero order than first order, we conclude that such active regulation of repression was unlikely.

For clones 6 and 18, the decrease in cells producing fluorescence fit rather well to first-order kinetics (Figure 4b). Clone 6 in particular demonstrated a good fit to first-order kinetics and its R-squared values (Table 2) were 0.996 or greater in all experiments. However, clones 6 and 18 behaved differently when passaged with different splitting regimens. For clone 18, the rate of fluorescence loss increased as the cultures were replenished by greater fractions of medium (Figure 4c, right). Since here greater passaging fractions necessitated a greater frequency of cell division, it is possible that clone 18 stochastically represses GFP in a cell division-dependent manner. Such a mechanism might be similar to the cell cycle-mediated silencing that has been reported in yeast [23]. In contrast, for clone 6 the repression rate of GFP was for the most part unaffected by its splitting schedule and largely independent of number of cell generations (Figure 4c, middle). Unlike clone 18, the loss of fluorescent cells appeared to depend on time (days) alone. Because of its good fit to first-order kinetics, the rate constant for clone 6 is a direct measurement of the probability of repression. With a rate of 0.04 days^-1^, this meant that a cell highly expressing GFP had a 1 in 25 chance each day to become a cell with repressed GFP.

### *Cis*-acting site dependence of repression kinetics

**Figure 5:**
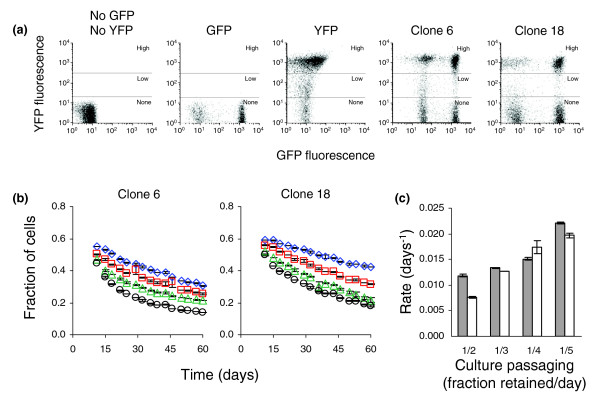
Repression of YFP by clones 6 and 18 with varied culture passaging conditions. **(a) **Levels of YFP fluorescence, high, low, and none, were defined by flow cytometry profiles of untransduced cells (No GFP, No YFP), cells transduced with GFP only, cells transduced with YFP only, and clones 6 and 18 transduced with YFP. **(b) **Fraction of cells with high YFP fluorescence. X-axis, time in days. When passaging cultures daily, different fractions of culture were retained: one-half (diamonds), one-third (squares), one-quarter (triangles), one-fifth (circles). **(c) **Rates of YFP repression for clones 6 (gray) and 18 (white).

Because the clones differ only by vector integration site (Table 1), it was the location in the genome that uniquely defined the rate and stability of transgene repression. But were these differences due to the local DNA environment? Or, did the integration event activate or deactivate some other trans-acting factor that globally affected transgene repression? We re-infected clones 6 and 18 with the same retroviral vector, but with GFP replaced by yellow fluorescent protein (YFP) (Figure 5a). To minimize effects from a single YFP integration site, we did not expand clones from a single cell; thus, from one culture, cells expressed YFP from numerous locations genome-wide. If the GFP integration affected a trans-acting factor, then that factor would affect the gene repression of YFP. But since clones 6 and 18 demonstrated little discernable difference in YFP repression (Figure 5b,c), it is likely that differences in GFP repression are due to *cis*-acting factors at the integration site. The repression of YFP was similar to that of GFP in clone 18, and the rate of YFP repression for clones 6 and 18 increased as the culture splitting increased. This suggests that it may be the norm for cell generation frequency to affect the transgene repression and, apparently, the stable kinetics of GFP repression in clone 6 is unique. Since its retroviral vector was integrated just 6 kb away from Brg1, a chromatin remodeling factor that affects transcription [24,25], we thought BRG1 expression could have been affected by the nearby integration and thus affect GFP expression. Yet we could discern no difference in expression of Brg1 protein after western blotting cell extracts (data not shown; here we probably would not have been able to distinguish between one and two alleles' worth of expression).

### Methylation and histone deacetylation

**Figure 6:**
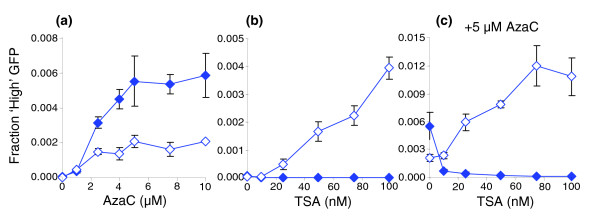
Inhibition of repression by azacytidine (AzaC) and trichostatin A (TSA) in clones 6 and 18. Fraction of clone 6 (filled diamonds) and clone 18 (open diamonds) cells with high GFP fluorescence after two days in the presence of **(a) **azacytidine, **(b) **trichostatin A, and **(c) **5 μM azacytidine plus trichostatin A.

Clones 6 and 18 were sorted for cells with repressed GFP - low and non-detectable populations, respectively. These cells were grown with two inhibitors of gene repression: azacytidine, which inhibits DNA methylation, and trichostatin A, which inhibits histone deacetylation. After two days of incubation with azacytidine, in both clones cells emerged that expressed a high level of GFP (Figure 6a), suggesting that methylation played a role in the repression of GFP. The reemergence of unrepressed clone 18 cells also occurred in the presence of trichostatin A alone (Figure 6b) and in combination with 5 μM azacytidine (Figure 6c), suggesting that histone deacetylation was also involved in GFP repression. In contrast, addition of trichostatin A to clone 6 did not yield any cells expressing a high level of GFP and it actually led to increased repression when in the presence of 5 μM azacytidine. These different responses of the clones to chemical inhibitors suggest differences in the mechanism of repression and further underscore how different mechanisms may be affected by genomic position. However, because the chemical inhibitors do not target only the transgene integration sites, we cannot make conclusions about the exact mechanisms that directly govern the observed gene repression.

## Discussion

We sought and found evidence that gene repression, which might appear sporadic, can lead to a highly reproducible outcome. We could demonstrate that the stochastic repression that accumulates over long periods of time can be described in terms of a probability. As much of our daily research involves the practice of cell culturing, we were impressed by the fact that a dynamic read-out could be largely independent of when and how cells were fed over a period of 80 days, and so we assign some importance to this observation. For a phenotype linked to the timed repression (or activation) of a single gene, an epigenetically based, stochastic timer might be well suited to dose the production of secreted factors, since the effective concentration of this factor depends on the collective secretion from a population of cells. Thus, it is possible that such a mechanism could schedule the dosage of hormones and drive the development of an embryo or child. Furthermore, because our study was performed using retroviral constructs, our observations may be immediately relevant to applications involving transgene expression with retroviruses, for example, retrovirus-mediated gene therapy.

In biology, the term 'stochastic' has been used with varying connotation and degrees of stringency; in all cases it means that there is an element of probability that is involved in a decision. If mice from a litter of congenic mice have different phenotypes, one might say that there is stochastic gene expression. Stochasticity has also been used to characterize natural fluctuations (up and down) in gene expression and this idea of stochasticity falls in line with the idea of an inherent level of 'noise.' Here, we have not studied 'noise', though no doubt this is also playing a role in our system. In an attempt to distinguish ourselves terminology-wise, we have tried to establish the idea of stochastic dynamics.

In conclusion, we investigated a system that behaves with near perfect first-order decay and by the inherent properties of a first-order system this strongly established that a stochastic decision was made during the gene repression. We showed that there can be locations in the genome where the repression is considerably less sensitive to, and perhaps even independent of, cell division frequency. We hope that our study will bolster the idea that in any biological system, as long as there is no instructional program, feed-back, or cellular quorum sensing, the observation of first-order gene repression is indicative of a stochastic mechanism.

## Materials and methods

### Vector construction

Constructs were based on the MoMLV retroviral vector contained in p102.21 (kindly provided by JB Lorens, Rigel Pharma, South San Francisco, USA). They contain enhanced green fluorescent protein (EGFP; Clontech, Mountatin View, CA, USA), or enhanced yellow fluorescence protein (EYFP; Clontech), followed by an internal ribosome entry site (IRES) and puromycin resistance gene. In plasmid pGFP-Eμ-IRES-Puro, EGFP was followed by the mouse Ig μ intron sequence, defined by a 1-kb Xba I fragment from the major intron. Plasmid pGFP-IRES-Puro contained no Ig enhancers.

### Cell culture

Retroviral vectors were packaged using PhoenixEco cells (ATCC SD 3444), and 18-81 mouse cells were infected with the vectors. All cultures were grown at 37°C and 5% CO2 in RPMI 1640 media with 10% fetal calf serum. Cells expressing GFP were isolated by FACS and clones were expanded from single cells. When used for expanding clones and monitoring gene repression, culture medium contained no puromycin. When selection was necessary, the medium contained 1.5 μg/ml puromycin. To vary the number of cell generations per day, cells were 'split' by different fractions. The passaging fractions and volumes (ml) in terms of culture plus pre-warmed fresh medium are: one-half, 1.5 + 1.5; one-third, 1.0 + 2.0; one-quarter, 0.75 + 2.25; one-fifth, 0.6 + 2.4; one-sixth, 0.5 + 2.5. In experiments where the fraction is not noted, cells were split by one-third. Cultures were split at the same time each day. Under these conditions, all cultures attained a saturation concentration of approximately 3 × 10^6^ cells/ml after one day. GFP expression was measured by flow cytometry, and for all analyses the relative fluorescence of cells without GFP was set to 3.0. Each flow cytometry measurement consisted of data from at least 5 × 10^3^ cells. The inhibitors of gene repression, azacytidine and trichostatin A, were added to the cultures in concentrations up to 100 μM and 10 nM, respectively.

### DNA sequencing

DNA from cells was isolated, and the GFP genes were PCR-amplified using Pfu polymerase (Stratagene, La Jolla, CA, USA). The PCR products were incubated with Taq polymerase to add deoxyadenosine overhangs and then cloned into pCR2.1-TOPO (Invitrogen, Carlsbad, CA, USA). The plasmids were then amplified in *E. coli *and sequenced.

### Quantitative RT-PCR

Expression of GFP and mouse beta-actin was measured using quantitative RT-PCR. RNA was extracted from cells and cDNA produced by standard application of reverse transcriptase and random hexamer primers. PCR was performed using the ABI PRISM^® ^7700 machine (Applied Biosystems, Foster City, CA, USA). GFP expression was normalized to the amount of expressed mouse beta-actin. Primers and probes were as follows: GFP, 5'-CCACATGAAGCAGCACGACT-3', 5'-TGCGCTCCTGGACGTAGC-3', 5'- [6-FAM]-TTCAAGTCCGCCATGCCCGAA- [TAMRA]-3'; beta-actin, 5'-AGGTCATCACTATTGGCAACGA-3', 5'-CACTTCATGATGGAATTGA-ATGTAGTT-3', 5'- [6-FAM]-TGCCACAGGATTCCATACCCAAGAAGG- [TAMRA]-3'.

### Identification of reporter integration sites

Integration sites of the vectors were PCR-amplified using Taq polymerase. The first amplification step of the PCR used a biotinylated primer homologous to the vector and a non-specific primer, degenerate at the 3' end and constant at the 5' end. Biotinylated PCR product was recovered by use of streptavidin-coupled magnetic beads (Invitrogen). The genomic DNA flanking the 3' long terminal repeat (LTR) of the provirus was then amplified from the biotinylated PCR product. This secondary PCR utilized a nested LTR-specific primer and a primer homologous to the constant section of the non-specific primer of the first PCR. PCR products were cloned into pCR2.1-TOPO, amplified in *E. coli*, and then sequenced. The final PCR product contained the virus-genome junction and the junction sequence was verified for each integration site.

## Additional data files

The following additional data are available with the online version of this paper. Additional data file 1 is a movie of GFP fluorescence of clone 5 over 84 days. Additional data file 2 is a movie of GFP fluorescence of clone 6 over 84 days. Additional data file 3 is a movie of GFP fluorescence of clone 18 over 84 days. Additional data file 4 contains additional flow cytometry data. Expression of GFP fluorescence as profiled by flow cytometry in clones 25 to 96. Clones 25 to 48 and 73 to 96 were transduced with GFP plus an adjacent Ig enhancer. Clones 1 to 24 (Figure 1b) and 49 to 72 were transduced with GFP without an Ig enhancer (Figure 1a). X-axis, relative fluorescence; Z-axis, normalized cell number; Y-axis, clones 25 to 39, (from front to back) after 13, 22, 32, and 42 days in culture; clones 40 to 48, after 22, 32, and 42 days; clones 49 to 96, after 13, 22, and 32 days; upper left corners show the clone identification number followed by the fraction of the population with fluorescence less than 100 after 32 days.

## Supplementary Material

Additional File 1 Movie of GFP fluorescence of clone 5 over 84 days. Click here for file

Additional File 2 Movie of GFP fluorescence of clone 6 over 84 days. Click here for file

Additional File 3 Movie of GFP fluorescence of clone 18 over 84 days. Click here for file

Additional File 4 Expression of GFP fluorescence as profiled by flow cytometry in clones 25 to 96. Clones 25 to 48 and 73 to 96 were transduced with GFP plus an adjacent Ig enhancer. Clones 1 to 24 (Figure 1b) and 49 to 72 were transduced with GFP without an Ig enhancer (Figure 1a). X-axis, relative fluorescence; Z-axis, normalized cell number; Y-axis, clones 25 to 39, (from front to back) after 13, 22, 32, and 42 days in culture; clones 40 to 48, after 22, 32, and 42 days; clones 49 to 96, after 13, 22, and 32 days; upper left corners show the clone identification number followed by the fraction of the population with fluorescence less than 100 after 32 days. Click here for file

## Figures and Tables

**Table 1:** Vector integration sites in clones

Clone	Chromosome	Base number	Orientation	Closest gene	Distance (bp)
2	4	119,028,655	+	*Hivep3*	in intron
3	3	95,092,146	+	*Mllt11*	347
5	16	76,364,060	-	*Nrip1*	in intron
6	9	21,506,165	-	*Smarca4*	5,878
7	10	62,465,169	+	Slc25a16	in exon
12	11	75,541,573	-	*Serpinf2*	20,524
13	11	118,056,600	+	*Socs3*	18,534
18	5	141,941,240	+	*Actb*	1,613
19	11	106,269,600	-	*Limd2*	417
24	2	158,459,470	+	*Ppp1r16b*	in intron

Chromosome, base number, and orientation (+/-) correspond to positions defined by the University of California, Santa Cruz Genomics Bioinformatics database for the mouse (August 2005 assembly). The closest known gene to the integration site and its distance to the site are also given.

**Table 2:** R-squared correlation coefficients for GFP repression data fit to zero-, first-, or second-order kinetics

Passaging	Clone								
	
	5			6			18		
	
	0th	1st	2nd	0th	1st	2nd	0th	1st	2nd
One-half	0.997	0.980	0.943	0.859	0.996	0.820	0.989	0.981	0.961
One-third	0.955	0.940	0.909	0.876	0.999	0.847	0.962	0.989	0.992
One-quarter	0.883	0.906	0.891	0.861	0.997	0.796	0.972	0.992	0.975
One-fifth				0.871	0.999	0.881	0.966	0.991	0.995
One-sixth							0.934	0.990	0.963

R-squared correlation coefficients for GFP repression data (Figure 4a) fit to zero, first (Figure 4b), or second-order kinetics. Values are shown for clones 5, 6, and 18, passaged daily with different fractions of culture retained.
