# Supplementary figures and images for: Slow, stochastic transgene repression with properties of a timer
Source: Genome Biol. 2006 Jun 9;7(6):R47. doi: 10.1186/gb-2006-7-6-r47 (PMC1779533; doi:10.1186/gb-2006-7-6-r47)

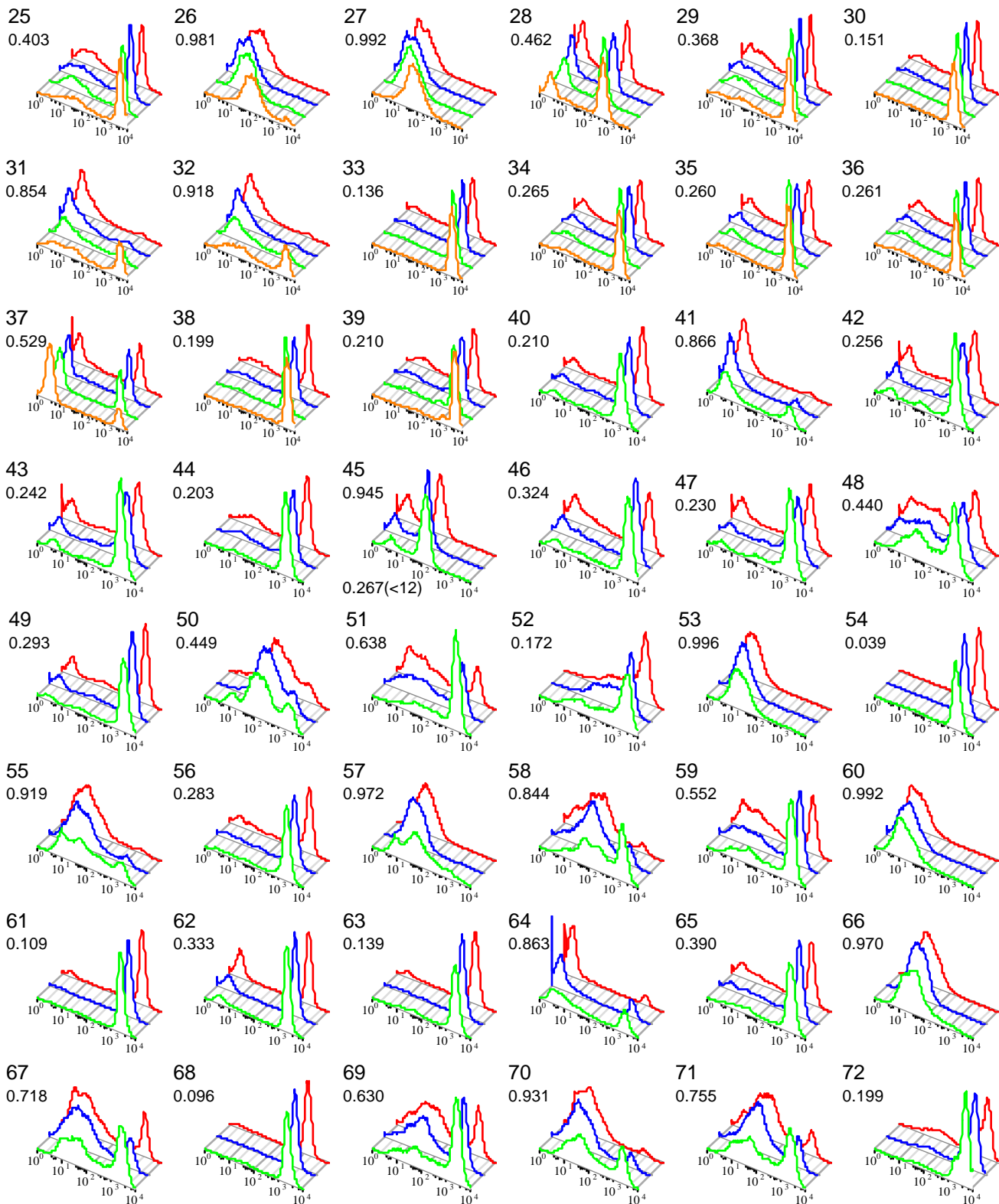

Additional flow cytometry data  
Wang, CL 2006

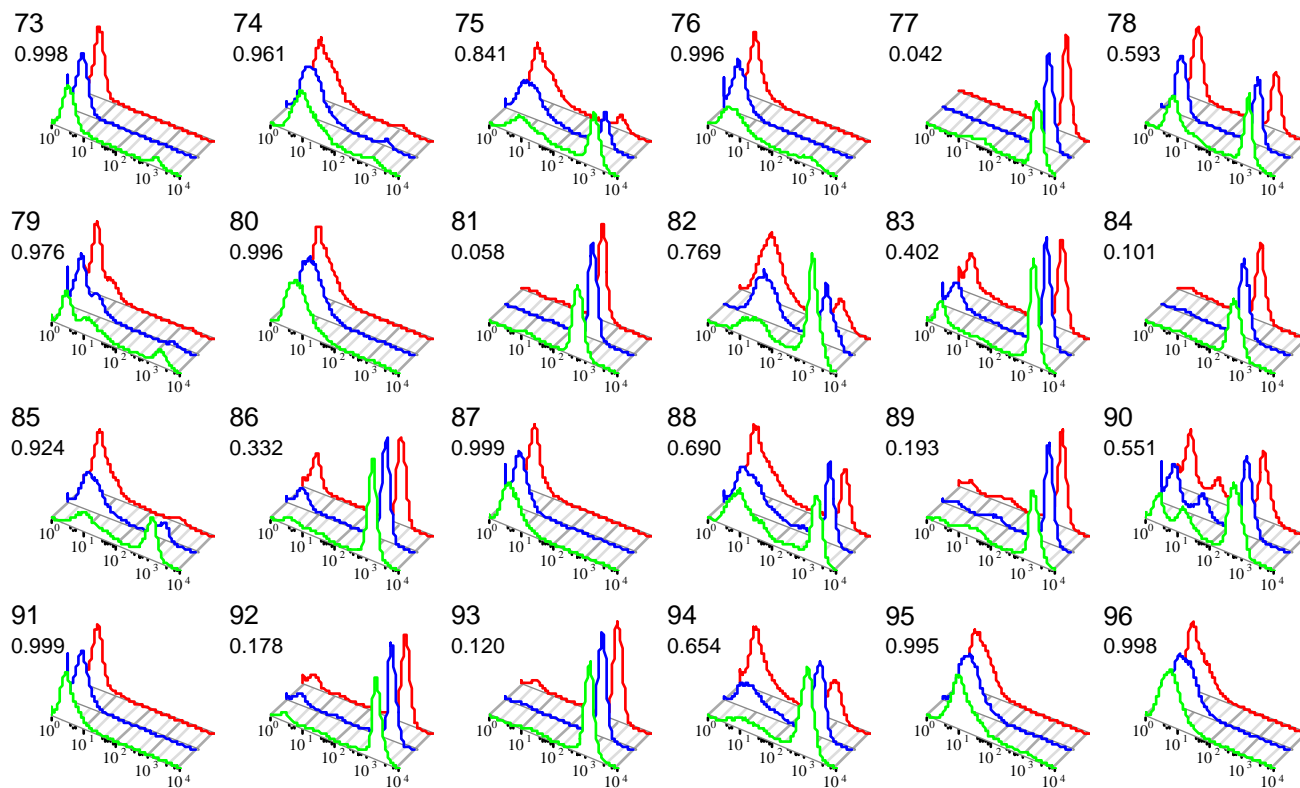

Additional flow cytometry data (continued)  
Wang, CL 2006

Supplement: Additional File 4 — Expression of GFP fluorescence as profiled by flow cytometry in clones 25 to 96. Clones 25 to 48 and 73 to 96 were transduced with GFP plus an adjacent Ig enhancer. Clones 1 to 24 (Figure 1b) and 49 to 72 were transduced with GFP without an Ig enhancer (Figure 1a). X-axis, relative fluorescence; Z-axis, normalized cell number; Y-axis, clones 25 to 39, (from front to back) after 13, 22, 32, and 42 days in culture; clones 40 to 48, after 22, 32, and 42 days; clones 49 to 96, after 13, 22, and 32 days; upper left corners show the clone identification number followed by the fraction of the population with fluorescence less than 100 after 32 days. [file gb-2006-7-6-r47-S4.pdf]
